# *IL-17F* Gene rs763780 and *IL-17A* rs2275913 Polymorphisms in Patients with Periodontitis

**DOI:** 10.3390/ijerph18031081

**Published:** 2021-01-26

**Authors:** Małgorzata Mazurek-Mochol, Małgorzata Kozak, Damian Malinowski, Krzysztof Safranow, Andrzej Pawlik

**Affiliations:** 1 Department of Periodontology, Pomeranian Medical University, 70-111 Szczecin, Poland; malgorzata.mazurek@poczta.onet.pl; 2 Department of Prosthetics, Pomeranian Medical University, 70-111 Szczecin, Poland; gosia-ko@o2.pl; 3 Department of Pharmacology, Pomeranian Medical University, 70-111 Szczecin, Poland; damian.malinowski@pum.edu.pl; 4 Department of Biochemistry and Medical Chemistry, Pomeranian Medical University, 70-111 Szczecin, Poland; chrissaf@mp.pl; 5 Department of Physiology, Pomeranian Medical University, 70-111 Szczecin, Poland

**Keywords:** IL-17, gene, polymorphism, periodontitis

## Abstract

*Background*: Periodontitis (PD) is a chronic inflammatory disease that can eventually lead to tooth loss. Genetic and environmental factors such as smoking are involved in the pathogenesis of PD. The development of PD is potentiated by various pathogens that induce an immune response leading to the production of cytokines, such as interleukin (IL)-17. The synthesis of IL-17 is influenced genetically. The polymorphisms in *IL-17* gene may affect the synthesis of IL-17. The aim of this study was to examine the association between the *IL-17F* rs763780 and *IL-17A* rs2275913 polymorphisms and PD in non-smoking and smoking patients to check if these polymorphisms could be a risk factor for PD. *Methods*: The study enrolled 200 patients with PD (130 non-smokers and 70 smokers) and 160 control subjects (126 non-smokers and 34 smokers). Periodontitis was diagnosed on the basis of 2017 World Workshop on the Classification of Periodontal and Peri-Implant Diseases and Conditions. All samples were genotyped using allelic discrimination assays with TaqMan^®^ probes on a Real-Time PCR Detection System. *Results*: There were no statistically significant differences in the distribution of the *IL-17F* rs763780 and *IL-17A* rs2275913 genotypes and alleles between patients with PD and control subjects, between smoking patients with PD and smoking control subjects, and between non-smoking patients with PD and non-smoking control subjects. *Conclusions*: The results of this study suggest a lack of statistically significant associations between *IL-17F* rs763780 and *IL-17A* rs2275913 polymorphisms and PD in Polish population.

## 1. Introduction

Periodontitis (PD) is characterized by inflammation and infiltration of inflammatory cells in periodontal tissue and continues to be a major public health problem [1]. Previous studies have shown that PD is associated with other diseases such as coronary artery disease, rheumatoid arthritis and diabetes, therefore early diagnosis and treatment of this disease is so important [2]. The pathogenesis of PD is multifactorial. This disease is related to bacterial infections that induce an immune response [3,4]. Susceptibility to PD may be caused by genetic factors as well as environmental factors, such as smoking and poor oral hygiene [1]. In periodontal tissue, various factors induce an immune response leading to the production of inflammatory mediators, including proinflammatory cytokines, and subsequently to tissue destruction [5,6,7,8]. Tobacco smoking is a very important risk factor for PD development. Smoking has been shown to affect the inflammatory response and synthesis of cytokines involved in inflammation in PD [9,10,11,12,13,14,15]. 

Interleukin (IL)-17 is a proinflammatory cytokine involved in the pathogenesis of PD and other inflammatory diseases [16]. This cytokine is produced by CD + T helper, hematopoietic cells, Th17 cells and neutrophils and consists of a family of cytokines from IL-17A to IL-17F. The IL-17 family plays a significant role in host defence against bacteria [17,18,19]. IL-17, with tumour necrosis factor-α and IL-1β, induces the synthesis of pro-inflammatory mediators in fibroblasts and keratinocytes leading to tissue inflammation [20,21,22].

The interplay between environmental and genetic factors induces the development of PD [1]. Previous studies indicated that the production of cytokines is genetically determined [23,24,25]. Due to the genetic polymorphisms of cytokine genes, individuals can be divided into 3 groups—dominative homozygotes producing increased amounts of cytokine, recessive homozygotes producing decreased amounts of cytokine, and heterozygotes producing intermediate amounts of cytokine. These inter-individual differences in cytokine production may influence the immune response to bacterial infection in periodontal tissues. 

Genes encoding IL-17A and IL-17F are located on chromosome 6 (6p12) [17]. In *IL-17* genes functional polymorphisms (*IL-17F* rs763780 and *IL-17A* rs2275913) that alter mRNA and protein expression has been found [26]. Polymorphisms of these genes were studied in various diseases with an immune background. These polymorphisms were associated with susceptibility to several diseases, including psoriasis, ulcerative colitis, rheumatoid arthritis, atopic asthma, immune thrombocytopenia [26,27,28,29,30,31]. Factors predisposing to PD, including cytokine gene polymorphisms, are currently being sought [32,33,34,35]. Cytokine gene polymorphisms were studied in various populations as the risk factors for PD in smoking and non-smoking patients [23]. Unfortunately, the results of studies are inconsistent. In this study, we examined the polymorphisms of *IL-17F* rs763780 and *IL-17A* rs2275913 in PD patients to see if they could be a risk factor for the development of this disease in Polish population.

## 2. Material and methods

### 2.1. Study Subjects

The patients from the West Pomeranian region of Poland, who came in years 2017–2019 to the Department of Periodontology with periodontitis had a medical interview and also a clinical and periodontal examination. Periodontitis was diagnosed on the basis of the 2017 World Workshop on the Classification of Periodontal and Peri-Implant Diseases and Conditions [36]. 

This study included 360 Caucasian subjects: 200 patients with chronic periodontitis (84 men, 116 women, aged 26–69 years, mean 50.47 ± 9.09, 130 were non-smokers and 70 were smokers) and 160 healthy subjects without periodontal disease as a control group (61 men, 99 women, aged 25–69 years, mean 42.97 ± 11.22, 126 were non-smokers and 34 were smokers). 

Autoimmune diseases and diseases such as hepatitis, AIDS, diabetes or uncontrolled hypertension excluded patients from the study. Also the patients who used systemic antimicrobial agents, chronic anti-inflammatory medication, immunosuppressive medications and antibiotic within the past 6 months were excluded. All patients were otherwise healthy and had not been subjected to periodontal treatment before the study.

The subjects were categorized into four subgroups: with and without periodontitis, according to the presence of smoking addiction (patients with periodontitis additionally smoking, patients with periodontitis with no smoking addiction, patient with healthy periodontium with or without smoking addiction). Smokers included people who had been continuously smoking at least 10 cigarettes a day for at least 5 years. 

The study was approved by the ethics committee at Pomeranian Medical University, Szczecin (number BN-001/93/08), Poland. All participants gave informed written consent to participate in the study. 

### 2.2. Periodontal Examination

Clinical attachment loss (CAL) and probing pocket depth (PPD) were performed in each patient at six sites per tooth using a periodontal probe marked every 1 mm (Hu-Friedy Mfg Co Inc, Chicago, IL, USA). Pressure of approximately 20 g was applied for probing. In addition, approximal plaque index (API) and modified sulcus bleeding index (mSBI) were calculated. Clinical measurements were performed by a periodontist specialist in each patient qualified for the study in the same conditions in a dental clinic—based on periodontal examination using the same instruments (periodontal probe calibrated every 1 mm, Nabers probe, periotest) and radiological examination (orthopantomogram). 

Periodontitis was detected as clinical attachment loss with a standardized periodontal probe with reference to the cemento-enamel junction. Patients were diagnosed with periodontitis if interdental CAL ≥ 2 was detectable at ≥2 non-adjacent teeth or buccal or oral CAL ≥ 3 mm and pockets > 3 mm were detectable at ≥2 teeth and the observed CAL couldn’t be attributed to non-periodontal causes. Radiographic bone loss had to be at least 15%. Extent and distribution of periodontitis was described as generalized when more than 30% of teeth were involved. 

### 2.3. Genotyping

All samples were genotyped in duplicate using allelic discrimination assays with TaqMan^®^ probes (Applied Biosystems, Carlsbad, CA, USA) on a 7500Fast Real-Time PCR Detection System (Applied Biosystems, Carlsbad, CA, USA).

### 2.4. Statistical Analysis

The consistency of the genotype distribution with Hardy–Weinberg equilibrium (HWE) was assessed with Fisher’s exact test. Chi-square and Fisher’s exact tests were used to compare genotype and allele distributions between groups. A *p*-value of < 0.05 was considered to indicate a statistically significant result. The study sample size was sufficient to detect with 80% probability the true effect size for comparison of allele frequencies between whole groups of PD patients and controls measured as odds ratio (OR) equal to 0.64 or 1.53 for rs2275913 and 0.18 or 2.51 for rs763780. The corresponding minimal detectable effect sizes (OR values) for subgroups of subjects stratified according to smoking status were: 0.59 or 1.65 for rs2275913 and 0.09 or 2.79 for rs763780 in non-smokers, and 0.37 or 2.33 for rs2275913 and 5.89 for rs763780 in smokers.

## 3. Results

The clinical periodontal parameters in the studied groups are shown in Table 1. We observed significantly increased values of approximal plaque index, sulcus bleeding index, probing pocket depth and clinical attachment loss in patients with periodontitis.

The distribution of the *IL-17F* rs763780 and *IL-17A* rs2275913 genotypes among smoking and non-smoking patients with PD as well as smoking and non-smoking control subjects was in HWE and is shown in Table 2. The distribution of *IL-17F* rs763780 and *IL-17A* rs2275913 genotypes and alleles did not differ between patients with periodontitis and controls. 

**Figure 1:**
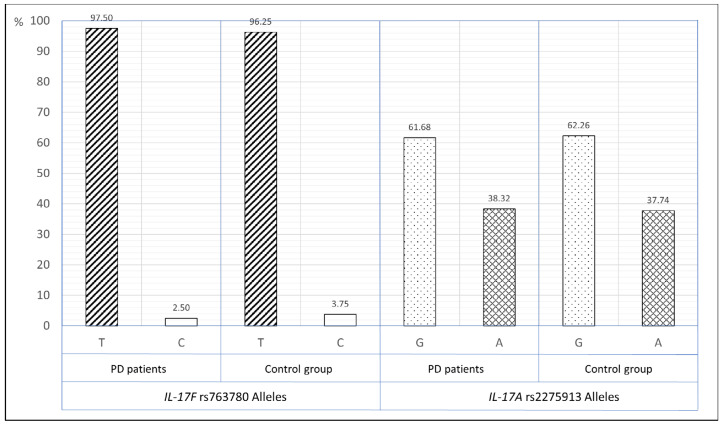
The distribution of *IL-17F* rs763780 and *IL-17A* rs2275913 alleles in periodontitis patients and control group.

The frequency of *IL-17F* (rs763780) T and C alleles in total group of PD patients was 97.50% and 2.50% respectively and did not differ statistically significant from the control group (96.25% and 3.75%), OR—0.66, 95%CI—0.28–1.54, *p* = 0.39, (Figure 1).

The frequency of *IL-17A* (rs2275913) G and A alleles in total group of PD patients was 61.68% and 38.32% respectively and did not differ statistically significant from the control group (62.26% and 37.74%), OR—1.03, 95%CI—0.76–1.39, *p* = 0.88, (Figure 1).

Since smoking can affect cytokine synthesis we also compared the distribution of studied polymorphisms between smoking patients with PD and smoking control subjects, and between non-smoking patients with PD and non-smoking control subjects. As shown in Table 3 and Table 4 these differences were not statistically significant.

**Figure 2:**
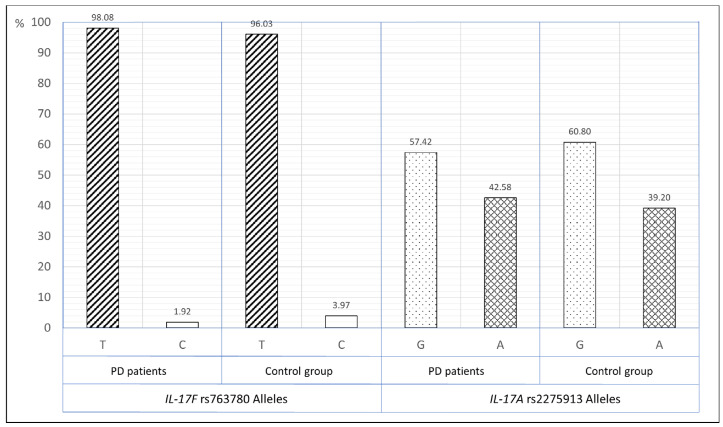
The distribution of *IL-17F* rs763780 and *IL-17A* rs2275913 alleles in periodontitis patients and control group in non-smokers group.

The frequency of the *IL-17F* (rs763780) T and C alleles was 98.08% and 1.92%, respectively in non-smoking patients with PD, while in the non-smoking control group it was 96.03% and 3.97% respectively (OR—0.48, 95%CI—0.16–1.41). In non-smoking patients with PD, the frequency of the *IL-17A* (rs2275913) G and A alleles was 57.42% and 42.58%, respectively, while in the non-smoking control group it was 60.80% and 39.20%, respectively (OR—1.15, 95%CI—0.81–1.64), (Figure 2).

**Figure 3:**
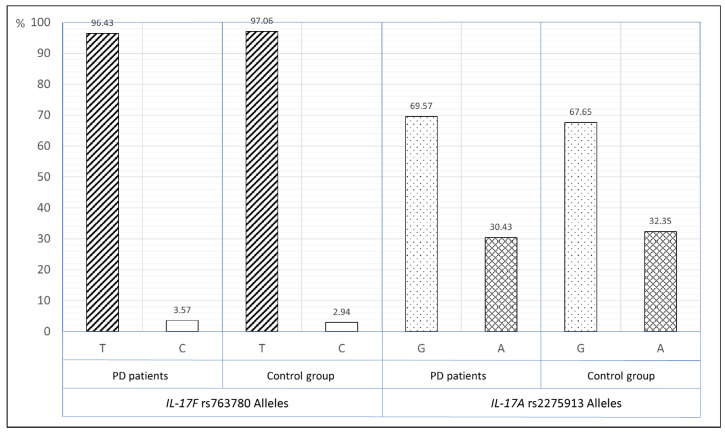
The distribution of *IL-17F* rs763780 and *IL-17A* rs2275913 alleles in periodontitis patients and control group in smokers group.

The frequency of the *IL-17F* (rs763780) T and C alleles in smoking patients with PD was 96.43% and 3.57%, respectively, while in the smoking control group it was 97.06% and 2.94%, respectively (OR—1.22, 95%CI—0.23–6.47). The frequency of *IL-17A* (rs2275913) G and A alleles in smoking PD patients was 69.57% and 30.43% respectively and did not differ significantly from the control group (67.65% and 32.35% respectively, OR—0.92, 95%CI—0.49–1.71), (Figure 3).

## 4. Discussion

In this study, we examined the relationship between the *IL-17F* rs763780 and *IL-17A* rs2275913 polymorphisms and PD to verify if these polymorphisms could be the risk factors for PD development. This analysis was performed separately in smoking and non-smoking patients and the control subjects. Tobacco smoking is one of the most important environmental risk factors for PD. Smoking may affect the host immune response to bacterial infections as well as the synthesis of proinflammatory mediators including cytokines [9,10,11,12,13,14,15]. Our results suggest a lack of statistically significant associations between these polymorphisms and PD both in smoking and non-smoking patients. In non-smoking PD patients we observed decreased frequency of *IL-17F* (rs763780) C allele (OR—0.48) and increased frequency of *IL-17A* (rs2275913) A allele (OR—1.15). In the group of smoking patients, the results were opposite. We observed increased frequency of *IL-17F* (rs763780) C allele (OR—1.22) and decreased frequency of *IL-17A* (rs2275913) A allele (OR—0.92). These results suggest that the effect of *IL-17* gene polymorphisms on PD risk may depend on smoking status. The pathogenesis of PD is very complex and involves interplay between genetic and environmental factors. It is likely that smoking may significantly alter the genetically determined synthesis of cytokines and the inflammatory response to bacterial infections in the periodontal tissues. 

IL-17 is a cytokine with a multi-directional action. It is involved in maintaining of barrier integrity in periodontal tissue and defence against pathogens. IL-17 induces the synthesis of antimicrobial mediators. On the other hand, these mediators enhance the inflammatory status in periodontal tissue and induce the development of periodontitis. Previous studies indicated the role of IL-17 in pathogenesis of PD. The studies have shown both increased and decreased expression of IL-17 in periodontal tissue in PD patients [37,38,39,40,41,42]. 

Several studies have investigated *IL-17* gene polymorphisms in patients with PD and the results are inconsistent. Saraiva et al. suggested that the *IL-17A* (rs2275913) A allele is associated with the lower frequency of PD in the Brazilian population [34]. Corrêa et al. [43] showed that the *IL-17A* (rs2275913) A allele is associated with an increased risk of chronic PD. In addition, the *IL-17A* (rs2275913) A allele was correlated with worse clinical parameters, higher myeloperoxidase activity, and increased expression of inflammatory mediators, compared with the other genotypes. Zacarias et al. [44] examined the association between *IL-17A* rs2275913 and *IL-17F* rs763780 polymorphisms and PD in the Brazilian population. These authors showed that the *IL-17A* (rs2275913) AA genotype and the A allele were associated with increased susceptibility to chronic PD. In another study, Chaudhari et al. [45] indicated that the *IL-17A* gene rs227591 polymorphism was associated with chronic and aggressive PD in the Indian population. The study by Jain et al. [46] did not confirm the association between the *IL-17F* gene rs763780 polymorphism and PD in the Indian population. Borilova et al. [47] examined the association between the *IL-17F* rs763780 and *IL-17A* rs2275913 polymorphisms and PD in patients with type 1 diabetes. The *IL-17A* (rs2275913) A allele was associated with an increased production of IL-17 by mononuclear cells of patients with PD. In a meta-analysis, da Silva et al. assessed the association between *IL-17F* rs763780 and *IL-17A* rs2275913 polymorphisms and the risk of chronic and aggressive PD [48]. This meta-analysis showed a lack of statistically significant associations between these polymorphisms and the risk of chronic and aggressive PD.

The observed differences between above studies may be due to ethnic differences, different forms and stages of PD as well as smoking status of patients. We have not indicated statistically significant associations between *IL-17* gene polymorphisms and the risk of PD. Our study is limited by the number of subjects. It is likely that these relationships could reach statistical significance in a larger cohort of patients. Our results suggest that the association between *IL-17* gene polymorphisms and PD may depend on smoking status. The interaction between genetic and environmental factors leads to inflammation in periodontal tissue. Smoking may affect the neutrophil activity, cytokine synthesis and inflammatory status [9,10,11,12,13,14,15]. Cytokine gene polymorphisms also may influence the cytokine production in response to bacterial infections and may be the risk factors for PD. However, it depends on the influence of many environmental factors, such as smoking. 

## 5. Conclusions

The results of this study suggest a lack of statistically significant associations between *IL-17F* rs763780 and *IL-17A* rs2275913 polymorphisms and PD in a Polish population. This association may depend on the smoking status of patients.

## Figures and Tables

**Table 1:** The clinical periodontal parameters of studied subjects.

Parameter	Controls	Periodontitis Patients	*p*-Value (Control vs. Periodontitis)	Controls (Smokers)	Periodontitis Patients (Smokers)	*p*-Value (Smokers: Control vs. Periodontitis)	Controls (Non-Smokers)	Periodontitis Patients (Non-Smokers)	*p*-Value (Non-Smokers: Control vs. Periodontitis)
SEX	55/105	84/116	0.16 ^#^	12/22	30/40	0.53 ^#^	43/83	54/76	0.25 ^#^
(M/F)									
AGE	45.28 ± 10.15	49.85 ± 8.71	*p* < 0.0001 *	44.18 ± 10.21	48.94 ± 8.89	*p* = 0.0036 *	45.58 ± 10.15	50.33 ± 8.61	*p* < 0.0001 *
(mean years ± SD)									
API %	35.81 ± 20.66	72.98 ± 21.03	*p* < 0.0001 *	48.29 ± 25.35	83.03 ± 17.45	*p* < 0.0001 *	32.44 ± 17.88	68.42 ± 19.50	*p* < 0.0001 *
(mean ± SD)									
SBI %	6.53 ± 11.29	57.66 ± 25.45	*p* < 0.0001 *	6.56 ± 10.09	46.83 ± 25.27	*p* < 0.0001 *	6.52 ± 11.63	63.96 ± 22.93	*p* < 0.0001 *
(mean ± SD)									
PPD mm	1.63 ± 0.34	4.36 ± 1.19	*p* < 0.0001 *	1.74 ± 0.29	4.74 ± 1.17	*p* < 0.0001 *	1.60 ± 0.35	4.15 ± 1.16	*p* < 0.0001 *
(mean ± SD)									
CAL mm	0.41 ± 0.92	5.06 ± 1.55	*p* < 0.0001 *	0.54 ± 0.96	5.30 ± 1.73	*p* < 0.0001 *	0.37 ± 0.91	4.93 ± 1.43	*p* < 0.0001 *
(mean ± SD)									

API—approximal plaque index, SBI—sulcus bleeding index, PPD—probing pocket depth, CAL—clinical attachment loss, * Mann-Whitney U test, ^#^ Fisher’s exact test (2-sided *p*-value).

**Table 2:** The distribution of *IL-17F* rs763780 and *IL-17A* rs2275913 genotypes in periodontitis patients and control group.

Genotype/Allele	PD Patients		Control Group		*p* ^a^	Compared Genotypes/Alleles	*p* ^b^	OR (95%CI)
	n	%	n	%				
*IL-17F* rs763780								
genotype								
TT	190	95.00%	148	92.50%	0.38			
TC	10	5.00%	12	7.50%		TC vs. TT	0.38	0.65 (0.27–1.54)
CC	0	0.00%	0	0.00%				
*IL-17F* rs763780								
Allele								
T	390	97.50%	308	96.25%				
C	10	2.50%	12	3.75%		C vs. T	0.39	0.66 (0.28–1.54)
*IL-17A* rs2275913								
genotype								
GG	81	41.12%	64	40.25%	0.82	AA + GA vs. GG	0.91	0.97 (0.63–1.48)
GA	81	41.12%	70	44.03%		AA vs. GA + GG	0.67	1.16 (0.66–2.03)
AA	35	17.76%	25	15.72%		AA vs. GG	0.76	1.11 (0.60–2.03)
						GA vs. GG	0.73	0.91 (0.58–1.45)
						AA vs. GA	0.65	1.21 (0.66–2.22)
*IL-17A* rs2275913								
Allele								
G	243	61.68%	198	62.26%				
A	151	38.32%	120	37.74%		A vs. G	0.88	1.03 (0.76–1.39)

^a^ χ^2^ test for *IL-17A* rs2275913 and Fisher’s exact test for *IL-17F* rs763780, ^b^ Fisher’s exact test, *IL-17F* rs763780, HWE: PD patients *p* = 1.0, control group *p* = 1.0, *IL-17A rs2275913*, HWE: PD patients *p* = 0.07, control group *p* = 0.50.

**Table 3:** The distribution of *IL-17F* rs763780 and *IL-17A* rs2275913 genotypes in periodontitis patients and control group in non-smokers group.

Genotype/Allele	PD Patients		Control Group		*p* ^a^	Compared Genotypes/Alleles	*p* ^b^	OR (95%CI)
	(Non-Smokers)		(Non-Smokers)					
	n	%	n	%				
*IL-17F* rs763780								
genotype								
TT	125	96.15%	116	92.06%	0.19			
TC	5	3.85%	10	7.94%		TC vs. TT	0.19	0.46 (0.15–1.40)
CC	0	0.00%	0	0.00%				
*IL-17F* rs763780								
Allele								
T	255	98.08%	242	96.03%				
C	5	1.92%	10	3.97%		C vs. T	0.20	0.48 (0.16–1.41)
*IL-17A* rs2275913								
genotype								
GG	44	34.38%	48	38.40%	0.75	AA + GA vs. GG	0.52	1.19 (0.71–1.99)
GA	59	46.09%	56	44.80%		AA vs. GA + GG	0.63	1.20 (0.63–2.28)
AA	25	19.53%	21	16.80%		AA vs. GG	0.59	1.30 (0.64–2.64)
						GA vs. GG	0.68	1.15 (0.66–1.99)
						AA vs. GA	0.86	1.13 (0.57–2.24)
*IL-17A* rs2275913								
Allele								
G	147	57.42%	152	60.80%				
A	109	42.58%	98	39.20%		A vs. G	0.47	1.15 (0.81–1.64)

^a^ χ^2^ test for *IL-17A* rs2275913 and Fisher’s exact test for *IL-17F* rs763780, ^b^ Fisher exact test, *IL-17F* rs763780, HWE: PD patients *p* = 1.0, control group *p* = 1.0, *IL-17A* rs2275913, HWE: PD patients *p* = 0.59, control group *p* = 0.57.

**Table 4:** The distribution of *IL-17F* rs763780 and *IL-17A* rs2275913 genotypes in periodontitis patients and control group in smokers group.

Genotype/Allele	PD Patients		Control Group		*p* ^a^	Compared Genotypes/Alleles	*p* ^b^	OR (95%CI)
	(Smokers)		(Smokers)					
	n	%	n	%				
*IL-17F* rs763780								
genotype								
TT	65	92.86%	32	94.12%	1.00			
TC	5	7.14%	2	5.88%		TC vs. TT	1.00	1.23 (0.23–6.69)
CC	0	0.00%	0	0.00%				
*IL-17F* rs763780								
Allele								
T	135	96.43%	66	97.06%				
C	5	3.57%	2	2.94%		C vs. T	1.00	1.22 (0.23–6.47)
*IL-17A* rs2275913								
genotype								
GG	37	53.62%	16	47.06%	0.65	AA + GA vs. GG	0.68	0.77 (0.34–1.75)
GA	22	31.89%	14	41.18%		AA vs. GA + GG	1.00	1.27 (0.37–4.39)
AA	10	14.49%	4	11.76%		AA vs. GG	1.00	1.08 (0.30–3.96)
						GA vs. GG	0.49	0.68 (0.28–1.66)
						AA vs. GA	0.74	1.59 (0.42–6.07)
*IL-17A* rs2275913								
Allele								
G	96	69.57%	46	67.65%				
A	42	30.43%	22	32.35%		A vs. G	0.87	0.92 (0.49–1.71)

^a^ χ^2^ test for *IL-17A* rs2275913 and Fisher’s exact test for *IL-17F* rs763780, ^b^ Fisher exact test, *IL-17F* rs763780, HWE: PD patients *p* = 1.0, control group *p* = 1.0, *IL-17A* rs2275913, HWE: PD patients *p* = 0.05, control group *p* = 0.71.

## List of Abbreviations

PD—periodontitis; IL—interleukin; PPD—probing pocket depth; CAL—clinical attachment loss; API—approximal plaque index; mSBI—modified sulcus bleeding index; HWE—Hardy–Weinberg equilibrium.

## References

[1] Kinane D.F., Peterson M., Stathopoulou P.G. (2006). Environmental and other modifying factors of the periodontal diseases. Periodontology 2000.

[2] Isola G., Polizzi A., Alibrandi A., Williams R.C., Leonardi R. (2020). Independent impact of periodontitis and cardiovascular disease on elevated soluble urokinase-type plasminogen activator receptor (suPAR) levels. J. Periodontol..

[3] Isola G., Polizzi A., Patini R., Ferlito S., Alibrandi A., Palazzo G. (2020). Association among serum and salivary A. actinomycetemcomitans specific immunoglobulin antibodies and periodontitis. BMC Oral Health.

[4] Sandros J., Karlsson C., Lappin D.F., Madianos P.N., Kinane D.F., Papapanou P.N. (2000). Cytokine responses of oral epithelial cells to Porphyromonas gingivalis infection. J. Dent. Res..

[5] Di Benedetto A., Gigante I., Colucci S., Grano M. (2013). Periodontal disease: Linking the primary inflammation to bone loss. Clin. Dev. Immunol..

[6] Isola G., Lo Giudice A., Polizzi A., Alibrandi A., Murabito P., Indelicato F. (2020). Identification of the different salivary Interleukin-6 profiles in patients with periodontitis: A cross-sectional study. Arch. Oral Biol..

[7] Mitani A., Niedbala W., Fujimura T., Mogi M., Miyamae S., Higuchi N., Abe A., Hishikawa T., Mizutani M., Ishihara Y. (2015). Increased expression of interleukin (IL)-35 and IL-17, but not IL-27, in gingival tissues with chronic periodontitis. J. Periodontol..

[8] Cheng W.C., van Asten S.D., Burns L.A., Evans H.G., Walter G.J., Hashim A., Hughes F.J., Taams L.S. (2016). Periodontitis-associated pathogens P. gingivalis and A. actinomycetemcomitans activate human CD14+ monocytes leading to enhanced Th17/IL-17 responses. Eur J. Immunol..

[9] Giannopolou C., Cappuyns I., Mombelli A. (2003). Effect of smoking on gingival crevicular fluid cytokine profile during experimental gingivitis. J. Clin. Periodontol..

[10] Persson L., Bergstrom J., Gustafsson A. (2003). The effect of tobacco smoking on neutrophil activity following periodontal surgery. J. Periodontol..

[11] Renvert S., Dahlén G., Wikstrom M. (1998). The clinical and microbiologicaleffects of non-surgical periodontal therapy in smokers and non-smokers. J. Clin. Periodontol..

[12] Persson L., Bergstrom J., Ito H., Gustafsson A. (2001). Tobacco smoking and neutrophil activity in patients with periodontal disease. J. Periodontol..

[13] Giannopoulos C., Kamma J., Mombelli A. (2003). Effect of inflammation, smoking and stress on gingival crevicular fluid level. J. Clin. Periodontol..

[14] Petropoulos G., McKay I., Hughes F. (2004). The association between neutrophil numbers and interleukin-1á concentrations in gingival crevicular fluid of smokers and non-smokers with periodontal disease. J. Clin. Periodontol..

[15] Bergström J. (2004). Tobacco smoking and chronic destructive periodontal disease. Odontology.

[16] Cheng W.C., Hughes F.J., Taams L.S. (2014). The presence, function and regulation of IL-17 and Th17 cells in periodontitis. J. Clin. Periodontol..

[17] Gaffen S.L. (2009). Structure and signalling in the IL-17 receptor family. Nat. Rev. Immunol..

[18] Miossec P., Kolls J.K. (2012). Targeting IL-17 and TH17 cells in chronic inflammation. Nat. Rev. Drug Discov..

[19] Zenobia C., Hajishengallis G. (2015). Basic biology and role of interleukin-17 in immunity and inflammation. Periodontology 2000.

[20] Gu C., Wu L., Li X. (2013). IL-17 family: Cytokines, receptors and signaling. Cytokine.

[21] Koenders M.I., Marijnissen R.J., Devesa I., Lubberts E., Joosten L.A., Roth J., van den Berg W.B. (2011). Tumor necrosis factor-interleukin-17 interplay induces S100A8, interleukin- 1 β, and matrix metalloproteinases, and drives irreversible cartilage destruction in murine arthritis: Rationale for combination treatment during arthritis. Arthritis Rheum..

[22] Iyoda M., Shibata T., Kawaguchi M., Hizawa N., Yamaoka T., Kokubu F., Akizawa T. (2010). IL-17A and IL-17F stimulate chemokines via MAPK pathways (ERK1/2 and p38 but not JNK) in mouse cultured mesangial cells: Synergy with TNF-alpha and IL-1beta. Am. J. Physiol. Renal. Physiol..

[23] Kozak M., Dabrowska-Zamojcin E., Mazurek-Mochol M., Pawlik A. (2020). Cytokines and Their Genetic Polymorphisms Related to Periodontal Disease. J. Clin. Med..

[24] Eerligh P., Koeleman B.P.C., Dudbridge F., Bruining G.J., Roep B.O., Giphart M.J. (2004). Functional genetic polymorphisms in cytokines and metabolic genes as additional genetic markers for susceptibility to develop type 1 diabetes. Genes Immun..

[25] Dziedziejko V., Kurzawski M., Paczkowska E., Machalinski B., Pawlik A. (2012). The impact of IL18 gene polymorphisms on mRNA levels and interleukin-18 release by peripheral blood mononuclear cells. Postepy. Hig. Med. Dosw..

[26] Tang H., Pei H., Xia Q., Tang Y., Huang J., Huang J., Pei F. (2017). Role of gene polymorphisms/haplotypes and serum levels of interleukin-17A in susceptibility to viral myocarditis. Mol. Biol. Rep..

[27] Nordang G.B.N., Viken M.K., Hollis-Moffatt J.E., Merriman T.R., Førre Ø.T., Helgetveit K., Kvien T.K., Lie B.A. (2009). Association analysis of the interleukin 17A gene in Caucasian rheumatoid arthritis patients from Norway and New Zealand. Rheumatology.

[28] Arisawa T., Tahara T., Shibata T., Nagasaka M., Nakamura M., Kamiya Y., Fujita H., Nakamura M., Yoshioka D., Arima Y. (2008). The influence of polymorphisms of interleukin-17A and interleukin-17F genes on the susceptibility to ulcerative colitis. J. Clin. Immunol..

[29] Tolba F.M., Diab S.M., Abdelrahman A.M.N., Behairy O.G., Almonaem E.R.A., Mogahed M.M., Mohamed S.A.-S. (2019). Assessment of IL-17F rs763780 gene polymorphism in immune thrombocytopenia. Blood Cells Mol. Dis..

[30] Liang T., Xu Y.T., Zhang Y., Cai P.C., Hu L.H. (2018). Interleukin-17A and -17F single nucleotide polymorphisms associate with susceptibility of asthma in Chinese Han population. Hum. Immunol..

[31] Kaur R., Rawat A.K., Kumar S., Aadil W., Akhtar T., Narang T., Chopra D. (2018). Association of genetic polymorphism of interleukin-17A & interleukin-17F with susceptibility of psoriasis. Indian J. Med. Res..

[32] Duarte P.M., Miranda T.S., Lima J.A., Dias Gonçalves T.E., Santos V.R., Bastos M.F., Ribeiro F.V. (2012). Expression of immune-inflammatory markers in sites of chronic periodontitis in patients with type 2 diabetes. J. Periodontol..

[33] Behfarnia P., Birang R., Andalib A.R., Asadi S. (2010). Comparative Evaluation of IFNγ, IL4 and IL17 Cytokines in Healthy Gingiva and Moderate to Advanced Chronic Periodontitis. Dent. Res. J..

[34] Saraiva A.M., Alves e Silva M.R.M., Correia Silva J.d.F., da Costa J.E., Gollob K.J., Dutra W.O., Moreira P.R. (2013). Evaluation of IL17A expression and of IL17A, IL17F and IL23R gene polymorphisms in Brazilian individuals with periodontitis. Hum. Immunol..

[35] Wang X., Zhang Y., Yang X.O., Nurieva R.I., Chang S.H., Ojeda S.S., Kang H.S., Schluns K.S., Gui J., Jetten A.M. (2012). Transcription of IL17 and IL17f is controlled by conserved noncoding sequence 2. Immunity.

[36] Caton J.G., Armitage G., Berglundh T., Chapple I.L.C., Jepsen S., Kornman K.S., Mealey B.L., Papapanou P.N., Sanz M., Tonetti M.S. (2018). A new classification scheme for periodontal and peri-implant diseases and conditions—Introduction and key changes from the 1999 classification. J. Clin. Periodontol..

[37] Johnson R.B., Wood N., Serio F.G., Johnson R.B. (2004). Interleukin-11 and IL-17 and the pathogenesis of periodontal disease. J. Periodontol..

[38] Stadler A.F., Angst P.D., Arce R.M., Gomes S.C., Oppermann R.V., Susin C. (2016). Gingival crevicular fluid levels of cytokines/chemokines in chronic periodontitis: A meta-analysis. J. Clin. Periodontol..

[39] Liukkonen J., Gürsoy U.K., Pussinen P.J., Suominen A.L., Könönen E., Liukkonen J. (2016). Salivary Concentrations of Interleukin (IL)-1beta, IL-17A, and IL-23 Vary in Relation to Periodontal Status. J. Periodontol..

[40] Honda T., Aoki Y., Takahashi N., Maekawa T., Nakajima T., Ito H., Tabeta K., Okui T., Kajita K., Domon H. (2008). Elevated expression of IL-17 and IL-12 genes in chronic inflammatory periodontal disease. Clin. Chim. Acta.

[41] Chitrapriya M.N., Rao S.R., Lavu V., Chitrapriya M.N. (2015). Interleukin-17 and interleukin-18 levels in different stages of inflammatory periodontal disease. J. Indian Soc. Periodontol..

[42] Cifcibasi E., Koyuncuoglu C., Ciblak M., Badur S., Kasali K., Firatli E., Cintan S. (2015). Evaluation of Local and Systemic Levels of Interleukin-17, Interleukin-23, and Myeloperoxidase in Response to Periodontal Therapy in Patients with Generalized Aggressive Periodontitis. Inflammation.

[43] Corrêa J.D., Madeira M.F.M., Resende R.G., Correia-Silva J.D.F., Gomez R.S., de Souza D.d.G., Teixeira M.M., Queiroz-Junior C.M., da Silva T.A. (2012). Association between polymorphisms in interleukin-17A and -17F genes and chronic periodontal disease. Mediat. Inflamm..

[44] Zacarias J.M., Sippert E.Â., Tsuneto P.Y., Visentainer J.E., de Oliveira e Silva C., Sell A.M. (2015). The Influence of Interleukin 17A and IL17F Polymorphisms on Chronic Periodontitis Disease in Brazilian Patients. Mediat. Inflamm..

[45] Chaudhari H.L., Warad S., Ashok N., Baroudi K., Tarakji B. (2016). Association of Interleukin-17 polymorphism (-197G/A) in chronic and localized aggressive periodontitis. Braz. Oral Res..

[46] Jain N., Joseph R., Balan S., Arun R., Banerjee M. (2013). Association of interleukin-4 and interleukin-17F polymorphisms in periodontitis in Dravidian ethnicity. Indian J. Hum. Genet..

[47] Borilova Linhartova P., Kastovsky J., Lucanova S., Bartova J., Poskerova H., Vokurka J., Fassmann A., Kankova K., Holla L.I. (2016). Interleukin-17A Gene Variability in Patients with Type 1 Diabetes Mellitus and Chronic Periodontitis: Its Correlation with IL-17 Levels and the Occurrence of Periodontopathic Bacteria. Mediat. Inflamm..

[48] Da Silva F.R.P., Pessoa L.D.S., Vasconcelos A.C.C.G., de Aquino Lima W., Alves E.H.P., Vasconcelos D.F.P. (2017). Polymorphisms in interleukins 17A and 17F genes and periodontitis: Results from a meta-analysis. Mol. Biol. Rep..

